# Views on Firearm Safety Among Caregivers of People With Alzheimer Disease and Related Dementias

**DOI:** 10.1001/jamanetworkopen.2020.7756

**Published:** 2020-07-15

**Authors:** Marian E. Betz, Deborah Azrael, Rachel L. Johnson, Christopher E. Knoepke, Megan L. Ranney, Garen J Wintemute, Daniel Matlock, Krithika Suresh, Matthew Miller

**Affiliations:** 1 Department of Emergency Medicine, School of Medicine, University of Colorado Anschutz Medical Campus, Aurora; 2 Veterans Affairs Eastern Colorado Geriatric Research Education and Clinical Center, Denver; 3 Harvard Injury Control Research Center, Harvard School of Public Health, Boston, Massachusetts; 4 Department of Biostatistics and Informatics, School of Public Health, University of Colorado Anschutz Medical Campus, Aurora; 5 Division of Cardiology, School of Medicine, University of Colorado Anschutz Medical Campus, Aurora; 6 Adult & Child Consortium for Outcomes Research & Delivery Science, School of Medicine, University of Colorado Anschutz Medical Campus; 7 Department of Emergency Medicine, Alpert Medical School, Brown University, Providence, Rhode Island; 8 Department of Emergency Medicine, University of California, Davis, Sacramento; 9 Division of Geriatric Medicine, Department of Medicine, University of Colorado School of Medicine, Aurora; 10 Department of Health Sciences, Bouvé College of Health Sciences, Northeastern University, Boston, Massachusetts

## Abstract

**Importance:**

The population of adults with Alzheimer disease and related dementias (ADRD) is increasing, and many people with ADRD have access to firearms. Little is known, however, about how caregivers of people with ADRD think about or address firearm safety.

**Objective:**

To assess views on firearm safety risks among caregivers of persons with ADRD, experiences of caregivers with health care professional–delivered counseling, and their preferred sources of information about firearm safety.

**Design, Setting, and Participants:**

This survey study used a probability-based online survey (National Firearms Survey, July 30 to August 11, 2019) with weights used to generate nationally representative estimates of adults living in households with firearms to assess firearm safety views of English-speaking adults 35 years or older. Respondents for the National Firearm Survery were drawn from I KnowledgePanel, a frame with approximately 55 000 US adults selected on an ongoing basis using address-based sampling methods.

**Main Outcomes and Measures:**

Caregivers were asked whether the person with ADRD owned or had access to firearms. Additional ADRD-related measures assessed perceptions about the likelihood of types of firearm injuries involving people with ADRD, support for firearm safety counseling by health care professionals with regard to dementia, and history of ever having received such counseling.

**Results:**

Of 6712 invited panel members, 4030 completed the survey (completion rate, 65%). For this analysis, we excluded the youngest participants (aged 18-34 years; n = 498); among the remaining 3532 participants, 124 reported being caregivers for persons with ADRD. Of the 124 caregivers, 51% were female and the mean (SD) age was 60 (12.5) years. Most participants (71%; 95% CI, 69%-72%) thought that a person with ADRD was more likely to hurt someone else unintentionally than intentionally hurt themselves or someone else. Many participants thought health care professionals should always (45%; 95% CI, 43%-47%) or sometimes (34%; 95% CI, 32%-37%) talk about firearm safety with caregivers or patients with dementia, but only 5% of caregivers (95% CI, 2%-12%) reported that a health care professional had ever spoken to them about firearm safety. Among the 41% (95% CI, 31%-51%) of caregivers who lived with a person with dementia, 31% (95% CI, 18%-49%) said the person with dementia could access firearms in the home.

**Conclusions and Relevance:**

In this study, few caregivers of adults with ADRD reported having received health care professional counseling about firearm safety, although most thought health care professionals should provide such counseling. These findings raise concerns about home firearm access among adults with ADRD and indicate potential opportunities for enhanced education by health care professionals and community organizations.

## Key Points

**Question:**

What proportion of adults living in homes with firearms are caregivers for people with Alzheimer disease and related dementias, and what are their characteristics, experiences, and preferences related to firearms?

**Findings:**

In this survey study of US adults (aged ≥35 years) living in homes with firearms, 2.6% reported being caregivers of a person with Alzheimer disease and related dementias, and 41% of these caregivers lived with that person. Although most caregivers were open to health care professional counseling about firearm safety for persons with Alzheimer disease and related dementias, few caregivers had ever received any such counseling.

**Meaning:**

The findings suggest that many community-dwelling adults with dementia have firearm access and that there may be a need for enhanced education and resources for their caregivers.

## Teaser

This survey study of caregivers of people with Alzheimer disease and related dementias assesses caregiver views on firearm safety risks, experiences with health care professional–delivered counseling, and preferred sources of information about firearm safety.

## Introduction

An estimated 39% to 49% of older adults live in a home with firearms.^1,2^ The proportions of these older adults who have dementia and of persons with dementia who have access to firearms are not well described. In a recent, small, nonrepresentative survey^3^ of caregivers of persons with dementia, one-quarter of the persons with dementia for whom they cared lived with firearms, and half of the caregivers identified firearm safety as a concern.

Despite the increasing population of older adults experiencing cognitive impairment and Alzheimer disease and related dementias (ADRD)^4^ and the potential for serious injury or death from firearm injuries (including firearm suicide),^5^ few research studies have examined firearm access among persons with dementia or firearm safety counseling for adult caregivers of persons with dementia. As such, existing guidelines for practitioners caring for persons with dementia, including recommendations about when and how to discuss firearm access with persons with dementia and their families,^5,6^ rely mostly on common sense and are not informed by specific information about firearm exposure among persons with dementia or caregiver perceptions and concerns related to firearm safety.

We aimed to expand on prior work^3^ by exploring firearm-related concerns and needs of caregivers of persons with dementia. We used the nationally representative 2019 National Firearms Survey (NFS) of adults living in homes with firearms. First, among these adults living in homes with firearms, we sought to compare caregivers with noncaregivers, hypothesizing that experiences with ADRD might be associated with firearm-related beliefs or behaviors. Second, we further examined caregivers’ knowledge, experiences, and preferences regarding firearm safety. The population of caregivers with personal experience with firearms (ie, those who are firearm owners or live in a home with firearms) was of interest because they may be more familiar with firearm safety concepts (vs caregivers without firearm experience). They may also be key trusted messengers for larger education campaigns to firearm owners, highlighting the importance of ensuring they are well informed about ADRD-associated risks and resources. Our results may inform future work in clinical and community interventions that are person centered and seek to enhance the safety of persons with dementia and those around them.

## Methods

### Design and Participants

This survey study used data from the online NFS conducted from July 30 to August 11, 2019, which was designed to examine firearm-related beliefs, behaviors, and ownership among a nationally representative sample of US adults living in homes with firearms. The Northeastern University Institutional Review Board approved the study with a waiver of written consent (ie, participants read informed consent language, and study completion constituted consent). All data were deidentified. This study followed the Strengthening the Reporting of Observational Studies in Epidemiology (STROBE) reporting guideline.

The investigators of NFS designed the survey and contracted the survey firm Ipsos (previously GfK) to administer it. Respondents were drawn from Ipsos’s KnowledgePanel, a frame with approximately 55 000 US adults selected on an ongoing basis using address-based sampling methods. KnowledgePanel members complete an annual panel survey on which household gun ownership status (along with numerous other characteristics, including household composition) is assessed, allowing for oversampling on these preidentified characteristics. KnowledgePanel is weighted to be representative of US adults, and a sample selection method is used to ensure that study samples behave as an equal probability of selection method.

The NFS targeted noninstitutionalized adults (aged ≥18 years) living in the US who were gun owners or nonowners living in a household with firearms excluding those in active US military service. Current household and personal gun status (owner vs nonowner in a household with firearms) was confirmed with specific questions in the 2019 NFS. Study-specific poststratification weights provided by Ipsos adjust for survey nonresponse and undercoverage or overcoverage imposed by the study-specific sample design. Study-specific poststratification weights also adjust for benchmark demographic distributions (from the US Census Current Population Survey or the American Community Survey) and, for population characteristics such as gun ownership (which are not available in the Current Population Survey or American Community Survey), from weighted KnowledgePanel profile data. For this study, the distributions of households with firearms from weighted KnowledgePanel data were used for the raking adjustment of weights, including sex, age, race/ethnicity, census region, metropolitan status, educational level, household income, and presence of children and adolescents younger than 18 years.

As with all KnowledgePanel surveys, Ipsos invited participants via email, with reminder emails sent to nonresponders on days 3, 6, 9, and 12. Ipsos has a modest point-based incentive program through which participants accrue points to redeem for cash, merchandise, or sweepstakes participation. Participants who completed the caregiver module described in this analysis received an extra cash equivalent of $5 from Ipsos.

For this study, we restricted the main analysis to adults 35 years or older who responded yes to the question, “Are you a currently a caregiver for an adult with Alzheimer disease or another kind of dementia? By caregiver, we mean someone (typically a family member or friend, sometimes with a power of attorney) who helps makes decisions for the person with dementia about medical, financial, housing or other needs.” We excluded the youngest participants (aged 18-34 years; n = 498) because of their low prevalence of caregiving. Of 6712 invited panel members, 4379 started and 4030 completed the larger survey (overall completion rate, 65%). For this analysis, we excluded the youngest participants (aged 18-34 years; n = 498); among the remaining 3532 participants, 124 reported being caregivers for persons with dementia.

### Measures

ADRD-related measures assessed views on the likelihood of different types of firearm injury (“accidentally harm self or someone else,” “injure self on purpose,” or “injure someone else on purpose,” each on a 3-point scale from least to most likely) and on whether health care professionals should discuss firearms if the patient or patient’s family member has ADRD. Additional questions related to ADRD were the participant’s relationship to the person with dementia; the person’s access to firearms and whether the participant had had concerns about this; whether the person with dementia owned firearms and, if so, had a plan for firearm transfer; and where the participant would look for if they had questions about firearm safety in the context of ADRD. Additional relevant main survey measures included caregiver age, sex, educational level, race/ethnicity, household characteristics (size, type, income, and living with the person with dementia), marital status, and region or rurality of residence, along with basic firearm use characteristics of the caregiver (carrying, frequency of use, and type of firearm owned).

### Statistical Analysis

Ipsos provided final survey weights that combined presample weights with study-specific poststratification weights to account for nonresponse and undercoverage or overcoverage imposed by the study-specific sample design. We conducted all analyses using Stata IC software, version 16 (StataCorp) and appropriate weighting commands (using the weight variable provided by Ipsos) to generate national estimates.

Frequency distributions and means were computed for participants’ demographic characteristics, experiences, and perspectives, with results presented as weighted proportions or medians with interquartile ranges. We performed χ^2^ tests and 2-sample *t* tests to assess associations between outcome variables and covariates (participant characteristics, experiences, and perspectives) within the framework of the weighted population. We conducted 2 stratified analyses. First, we compared covariates by caregiver status, hypothesizing that caregivers’ knowledge or experiences related to firearms might be different because of their experiences with dementia-associated cognitive and behavioral changes. Second, among caregivers, we compared covariates for those who lived with the persons with dementia with those who did not. Because all respondents lived in a home with 1 or more firearm (as an eligibility criterion for survey participation), the persons with dementia living with them also lived with 1 or more firearm. We hypothesized that caregivers who lived with the persons with dementia vs those who did not might report different rates of firearm access and ownership among the persons with dementia.

## Results

A total of 124 respondents (51% female; mean [SD] age, 60 [12.5] years) were caregivers to an adult with ADRD. Among US adults living in homes with firearms, 3% (95% CI, 2%-3%) reported being a caregiver for a person with dementia. Caregivers did not differ significantly from noncaregivers by demographic, socioeconomic, or most firearm-related factors. The only exception was that caregivers (19%; 95% CI, 12%-29%) (Table 1) were significantly more likely than noncaregivers (8%; 95% CI, 7%-9%) to report ever having stored a gun for a friend or family member for whom they were concerned it was not safe to have gun access; the survey did not systematically clarify whether the concern was for ADRD or another reason.

**Table 1.** Characteristics of the 124 Responding Caregivers

Characteristic	Finding^a^
Age group, y	
35-59	49 (39-59)
≥60	51 (41-61)
Female	51 (30-81)
Race/ethnicity	
Non-Hispanic white	80 (70-87)
Non-Hispanic black	10 (5-20)
Hispanic	3 (1-10)
Married	72 (62-81)
Household size, median (IQR)	2 (2-3)
Some college or higher	66 (55-75)
Carried loaded handgun in past 30 d	14 (9-23)
Frequency of handling gun for any reason in past y	
Daily, weekly, or monthly	17 (11-26)
Less than monthly	83 (74-89)
Personally owns firearms	
No, but lives with a gun owner	31 (23-41)
Yes	69 (59-77)
Handgun	85 (76-92)
Primarily for protection against people	82 (71-90)
Long gun	60 (48-71)
Primarily for hunting	45 (32-60)
Has had formal firearms training	46 (36-56)
Believes having a gun in the home makes it safer	83 (75-89)
Has ≥1 gun in home stored loaded and unlocked^b^	25 (16-37)
Has ever stored a gun for a friend or family member when concerned it was not safe for them to have access	19 (12-29)
As part of routine care, health care professionals should talk with their patients about firearms if their patient or patient's family member has ADRD	
Yes, always	55 (45-65)
Yes, sometimes	28 (20-37)
No	17 (10-26)

Abbreviations: ADRD, Alzheimer disease and related dementias; IQR, interquartile range.

^a^ Data are presented as weighted percentage (95% CI) unless otherwise indicated.

^b^ Estimate from owners.

**Figure 1.**
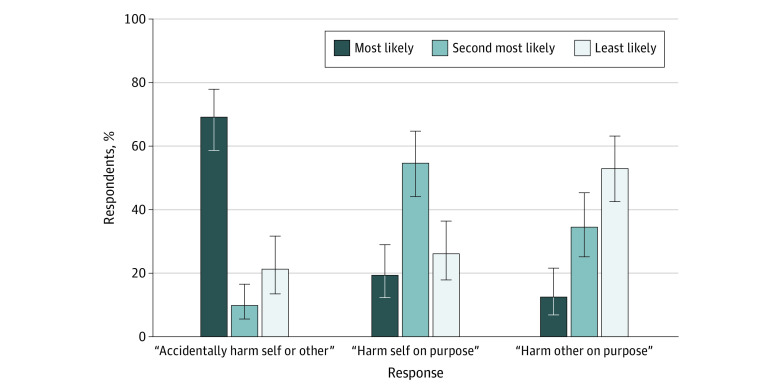
Rankings of Responses Among 124 Caregivers to the Question, “Which Is Most Likely for a Person With Alzheimer Disease?” Participants were asked to rank each of the 3 scenarios as most, second most, or least likely. Data are the aggregate responses with 95% CIs (error bars).

Participants were asked to rank the relative likelihood of various types of injury that might result from access to a gun by a person with ADRD (Figure 1); most caregivers said “accidentally harm self or someone else” was most likely (69%; 95% CI, 59%-78%), followed by “injure self on purpose” (19%; 95% CI, 15%-18%) and then “injure someone else on purpose” (13%; 95% CI, 7%-22%). Responses from noncaregivers were not significantly different, with 71% (95% CI, 69%-72%) saying “accidentally harm self or someone else” was most likely followed by 17% (95% CI, 15%-18%) saying “injure self on purpose” and 13% (95% CI, 12%-15%) saying “injure someone else on purpose.” For all respondents combined, 71% (95% CI, 69%-72%) said “accidentally harm self or someone else” was most likely, 17% (95% CI 16%-19%) said “injure self on purpose” was most likely, and 3% (12%-15%) said “injure someone else on purpose” was most likely.

When asked about the role of health care professionals, most respondents thought professionals should always (caregivers and noncaregivers combined: 45%; 95% CI, 43%-47%) or sometimes (caregivers and noncaregivers combined: 34%; 95% CI, 32%-37%) talk about firearms when patients or patients’ family members have ADRD. However, only 5% of caregivers (95% CI, 2%-12%) and 7% (95% CI, 6%-8%) of noncaregivers said a health care practitioner had ever spoken to them about firearm safety (Table 2).

**Table 2.** Characteristics and Experiences Among Caregivers, by Residence

Characteristic or experience	Weighted % (95% CI)		
	Total (N = 124)	Lives with person with dementia (n = 49)	Does not live with person with dementia (n = 75)
Person with dementia^a^			
Spouse or partner	17 (11-26)	36 (23-52)	4 (1-10)
Parent or stepparent	55 (45-65)	36 (22-54)	68 (55-79)
Another family member	18 (12-27)	22 (11-37)	17 (8-29)
Not related	9 (5-17)	6 (2-17)	12 (6-23)
Person with dementia able to access guns in your or their home^a^			
Yes	15 (9-24)	31 (18-49)	3 (1-10)
No, cannot any longer	25 (17-35)	18 (9-33)	30 (19-43)
No, have never been able to	60 (50-70)	50 (34-66)	67 (54-78)
Ever had concerns about person with dementia having firearm access	11 (7-19)	10 (4-23)	13 (7-23)
You, friends, or family did something	53 (28-77)	46 (12-85)	57 (26-83)
Does person with dementia own a firearm?^b^			
No	81 (72-88)	68 (51-81)	90 (80-95)
Not sure	2 (0.5-8)	2 (0.3-16)	2 (0.2-11)
Yes	17 (11-26)	29 (17-46)	9 (4-18)
Person with dementia has plan for securing, removing, or transferring guns if it becomes unsafe for them to handle them			
Yes, written	20 (7-45)	14 (3-46)	33 (7-75)
Yes, not written	38 (18-64)	33 (11-66)	51 (16-85)
No	23 (6-59)	32 (8-72)	0 (0-0)
Do not know or not sure	19 (6-48)	21 (5-58)	16 (2-63)
Health care practitioner has ever spoken to you about firearm safety	5 (2-12)	2 (0-11)	7 (2-18)

^a^ *P* < .001.

^b^ *P* < .01.

Among caregivers, 41% (95% CI, 31%-51%) lived with the person with dementia, more commonly when the person with dementia was a spouse or partner (Table 2). Nearly one-third (31%; 95% CI, 18%-49%) of caregivers living with a person with dementia said the person with dementia could access firearms in the home; only 3% of caregivers for persons with dementia living separately said the person with dementia could access firearms in the caregiver’s home (95% CI, 1%-10%). Overall, 11% (95% CI, 7%-19%) of caregivers reported ever having concerns about the person with dementia’s access to firearms; in half of those cases, the caregiver, friends, or family had taken some action.

Among all caregivers in the study, 17% (95% CI, 11%-26%) reported that the person with dementia personally owned a firearm; this prevalence was higher among caregivers living with the person with dementia (29%; 95% CI, 17%-46%) than among those living separately (9%; 95% CI, 4%-18%). Of these gun-owning persons with dementia, 58% (95% CI, 30%-81%) were reported to have some kind of plan for securing, removing, or transferring their guns in the event they became unsafe to handle them, although the plan was often not written (Table 2).

**Figure 2.**
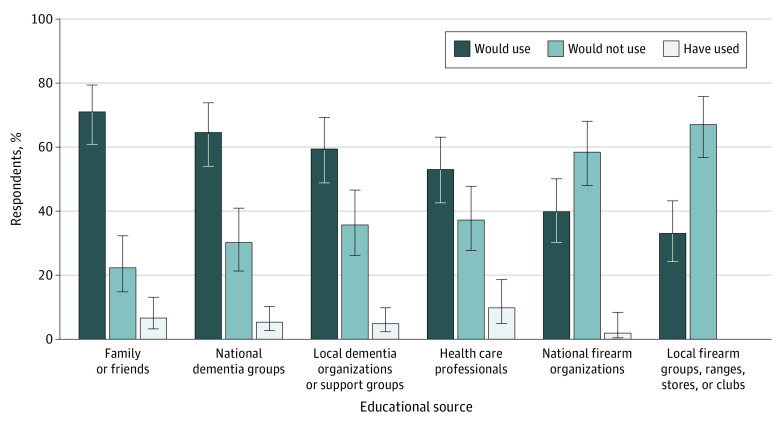
Responses of 124 Caregivers of Where They Had, Would, or Would Not Look for Answers to Questions About Firearm Safety in the Context of Dementia Multiple responses allowed. Error bars indicate 95% CIs.

Caregivers were asked where they had looked (or would look) for information about firearm safety in the context of dementia; among those who had looked for information, the most common source was health care professionals, followed by family or friends (Figure 2). In contemplating future sources of information, the greatest proportion said they would look to family or friends (72%; 95% CI, 30%-81%) or dementia-related organizations, either national (67%; 95% CI, 56%-76%) or local (61%; 95% CI, 51%-71%). Smaller yet notable proportions said they would look to firearm-related organizations at the national (40%; 95% CI, 31%-51%) or local (34%; 34%; 95% 25%-44%) level.

## Discussion

This nationally representative survey of adults living in homes with firearms revealed several key findings. Of importance, our results raise questions about home safety among person with dementia given that one-third of persons with dementia who lived with caregivers had access to home firearms. In addition, caregiver responses highlight misperceptions about relative risk among types of injury; responses also indicate potential opportunities for enhanced health care professional interactions and optimal sources for disseminating information. These findings, taken together, suggest the need for focused, person-centered clinical and community interventions to enhance safety of persons with dementia and those around them.

In this sample of adults 35 years or older living in homes with guns, 3% reported being a caregiver to a person with ADRD compared with an estimated 6% in the general adult US population in 2018.^7,8^ Contrary to our hypothesis that first-hand experiences with the cognitive and behavioral changes of ADRD might affect views or experiences, we found that caregivers did not differ from noncaregivers in certain key domains, such as views on whether a firearm makes a home safer and the role of health care professionals. Future work stratifying beliefs or experiences by the stage of dementia, specific behavioral symptoms, or the relationship between the caregiver and person with dementia may be helpful in developing targeted education for caregivers in homes with firearms.

Approximately 41% of caregivers of persons with dementia reported living with these individuals. Of note, among these caregivers, nearly one-third said the persons with dementia could access the firearms in the home. This finding was higher than among caregivers who lived separately from the persons with dementia, with only 3% of them indicating that the persons with dementia had firearm access at home; this finding may be because persons with dementia did not own firearms or were living in residential facilities where weapons were not allowed. Although this survey did not collect information on reported severity of dementia, our findings suggest the need for additional work. Two states explicitly prohibit firearm acquisition or carrying by persons with dementia,^6^ but otherwise there is little legal or policy guidance about what stage a person with dementia may no longer have access to firearms. Existing clinical guidelines generally recommend graduated restriction of access based on stage of dementia and presence of certain behavioral symptoms, such as paranoia or agitation, that can lead to violence.^5,6,9,10^

Among older adults as a general population, the highest risk of firearm injury is from self-harm.^11,12^ Suicide rates are high in the older adult population, especially among men, in large part because older adults are more likely to use firearms (and therefore have a lethal attempt).^13^ Rates of physical violence by persons with dementia toward others (not necessarily with a firearm) are approximately 24% for the entire course of illness, ranging from 18% to 64%, depending on the population and definition of violence.^14,15,16^ Among home-based caregivers of persons with dementia, approximately 20% have reported experiencing aggression or violence committed by the person with dementia.^14^ However, in our study, participants ranked intentional injury to others or unintentional harm to self or others as more likely than suicide, highlighting a critical knowledge gap.

Study findings underscore the role of health care professionals in addressing firearms in the home. Although most participants said health care professionals should discuss firearm safety in the context of ADRD, only 5% had ever had a health care professional talk with them about firearm safety for any reason. This finding suggests a significant gap in practice that may be associated with health care professional uncertainty or concerns about alienating patients, although prior work^16^ suggests that patients are open to respectful firearm safety counseling in appropriate contexts. Health care professional training, materials, and resources^16,17,18^ may facilitate more routine firearm safety counseling for persons with dementia and their families. When asked where they would turn for answers to firearm safety questions, caregivers identified health care professionals as an important source: 10% said they had already looked to them for help, and 53% said they would in the future. Official policies or guidance by health care organizations, such as the Veterans Health Administration,^17^ as well as templates to prompt and document counseling^19^ may encourage and support health care professionals.

In this sample, caregivers reported that many persons with dementia who owned firearms had no plan for what to do with the guns if their illness made it unsafe to handle them. Engaging firearm-owning persons with dementia early in the course of disease about planning for future firearm transfer may promote respect for independence and preferences while also ensuring safety. As with other topics (such as driving retirement),^20,21^ use of a firearm agreement may help prompt and document decisions.^6^ State laws for firearm sale or transfer vary, and clear, stepwise guidance for caregivers may be helpful. Engagement of the firearms community in disseminating educational material may also be useful because some caregivers identified firearms organizations or retailers as sources for information. Partnerships between dementia and firearm groups may be particularly helpful in broadly disseminating messages. One example is a collaboration between Alzheimer San Diego and a local firing range to provide education, guidance, and firearm locking devices.^22^ Future work is needed to examine the effects of these types of programs on beliefs, storage behaviors, and injury outcomes.

### Limitations

This study has limitations. This survey of caregivers did not directly assess the characteristics, beliefs, or behaviors of persons with dementia and did not confirm caregiver perceptions of persons with dementia’s ownership of or access to firearms. The study also relied on caregiver identification of Alzheimer disease or another form of dementia; thus, participants may have underreported caregiving for individuals with mild impairment. Future work engaging individuals with early stages of cognitive impairment or using objective methods for verification of home storage or access would be useful. Our definition of caregiver may have slanted the survey toward caregivers who are also (or primarily) surrogate decision-makers rather than those providing assistance in activities of daily living. This limitation, combined with the small sample size, suggests that our findings may underestimate the scope of the issue, especially as related to community-dwelling older adults with early cognitive impairment. The survey did not include questions about stage of dementia or whether persons with dementia lived in long-term facilities, and the sample size limited analyses in subgroups. In addition, we surveyed only adults living in homes with guns; thus, our results are not generalizable to the caregiver population as a whole.

## Conclusions

In this nationally representative survey study of caregivers of adults with ADRD living in homes with firearms builds on prior work^3^ by identifying specific concerns, needs, and misperceptions. When persons with dementia lived with the caregivers, nearly one-third were reported to have access to home firearms. Most caregivers ranked injury to others as more likely than self-injury, highlighting the need to raise awareness about the risk of firearm suicide among older adults and in the context of dementia. Experiences with health care professional counseling and desired sources of information indicate the need for further work to encourage routine firearm safety counseling and easy access to information and resources from trusted sources. Incorporating the views of key stakeholders, including caregivers, persons with dementia, firearm owners, and health care professionals, will be important to optimize safety and well-being of persons with dementia and those around them.
